# Self-selective van der Waals heterostructures for large scale memory array

**DOI:** 10.1038/s41467-019-11187-9

**Published:** 2019-07-18

**Authors:** Linfeng Sun, Yishu Zhang, Gyeongtak Han, Geunwoo Hwang, Jinbao Jiang, Bomin Joo, Kenji Watanabe, Takashi Taniguchi, Young-Min Kim, Woo Jong Yu, Bai-Sun Kong, Rong Zhao, Heejun Yang

**Affiliations:** 1 Department of Energy Science, Sungkyunkwan University, Suwon, 16419 Korea; 2 Singapore University of Technology & Design, 8 Somapah Road, 487372 Singapore, Singapore; 3 IBS Center for Integrated Nanostructure Physics (CINAP), Institute for Basic Science, Sungkyunkwan University, Suwon, 16419 Korea; 4 Department of Electrical and Computer Engineering, Sungkyunkwan University, Suwon, 16419 Korea; 5 National Institute for Materials Science, 1-1 Namiki, Tsukuba, 305-0044 Japan

**Keywords:** Electronic devices, Two-dimensional materials

## Abstract

The large-scale crossbar array is a promising architecture for hardware-amenable energy efficient three-dimensional memory and neuromorphic computing systems. While accessing a memory cell with negligible sneak currents remains a fundamental issue in the crossbar array architecture, up-to-date memory cells for large-scale crossbar arrays suffer from process and device integration (one selector one resistor) or destructive read operation (complementary resistive switching). Here, we introduce a self-selective memory cell based on hexagonal boron nitride and graphene in a vertical heterostructure. Combining non-volatile and volatile memory operations in the two hexagonal boron nitride layers, we demonstrate a self-selectivity of 10^10^ with an on/off resistance ratio larger than 10^3^. The graphene layer efficiently blocks the diffusion of volatile silver filaments to integrate the volatile and non-volatile kinetics in a novel way. Our self-selective memory minimizes sneak currents on large-scale memory operation, thereby achieving a practical readout margin for terabit-scale and energy-efficient memory integration.

## Abstract (web summary)

Designing large-scale crossbar arrays for energy efficient neuromorphic computing systems remains a challenge. Here, the authors propose Van der Waals (h-BN/graphene/h-BN) self-selective memory design able to combine, in the same cell, non-volatile and volatile behaviors with negligible sneak current.

## Introduction

The use of high-density, fast, and energy-efficient non-volatile memory (NVM) instead of the current flash memory technology has been explored for unprecedented applications, such as the internet of things and neuromorphic computing^1^. For this purpose, the concept of the crossbar array with a memory cell at each intersection has been considered the most optimal and promising architecture for more than 60 years^2^. Although various types of resistive memory cells between word lines and bit lines in the crossbar array architecture have been proposed^3–8^, a fundamental issue remains: the increasing role of interconnecting wire resistance and sneak currents in the memory array operation as we establish higher integration density (capacity). The most-studied solution to the above issue, one-selector one-resistor (1S1R) or one-transistor one-resistor (1T1R) array, still involves complex processes (particularly, current–voltage matching and etching fabrication problems) that are not compatible with three-dimensional (3D) integration^9,10^. Another important solution, complementary resistive switching memory, suffers from its destructive reading operation as well as its high off-current that causes the sneak current issue^11,12^. Therefore, a novel device architecture with self-selective memory function along with ultralow sneak currents, high selectivity and speed, and reliability are required.

Two-dimensional (2D) van der Waals heterostructures have provided various breakthroughs for material and device issues because of their unique stability and functionalities^13–19^. Recently, atomically-thin memory devices have been reported based on 2D transition metal dichalcogenides (TMDs);^20–22^ however, they do not have high self-selectivity, which prevents large-scale integration without using additional transistors. The reliability of TMDs-based memory devices remains an issue as well; the device performance differs from lab to lab^23^. As we pursue robust device operation with 2D materials, we paid attention to robust physics researches with 2D materials. Among the diverse 2D elements, two major building blocks, highly insulating hexagonal boron nitride (h-BN)^24^ and metallic graphene^25,26^, are chemically and mechanically stable, allowing easy stacking processes for vertical heterostructures and large-scale integration. Although the wafer-scale growths of 2D materials are challenging, a recent report on wafer-scale single crystal h-BN^27^ gives a promising opportunity for industrial applications of 2D materials. The relatively weak layer-to-layer interaction and strong in-plane atomic bonding nature of 2D h-BN and graphene could be used to realize an original self-selecting function that resolves the long-standing issues in the crossbar array.

We demonstrate a novel memory cell, a self-selective van der Waals heterostructure, constructed by stacking h-BN and graphene layers into a vertical structure of h-BN/graphene/h-BN between silver (Ag) and gold (Au) electrodes in a crossbar array structure. The unique roles of 2D materials (h-BN and graphene) in our self-selective memory can be described like below. First, the strong in-plane atomic bonding of high-quality h-BN layers provides a platform for ultralow off-state current and the endurance against high on-state currents (0.3 mA). Second, the strong in-plan atomic bonding of transferable graphene efficiently blocks the diffusion of Ag filaments, which can generate a voltage across the other h-BN layer without any Ag substance. With the assistance of graphene, our van der Waals heterostructure could demonstrate the volatile and non-volatile dynamics in one cell. We note that the graphene cannot be replaced by other typical metals because Ag filaments can be easily diffused through those metals. Accordingly, our self-selective 2D memory cell based on h-BN and graphene resolves the current–voltage matching and integration issue of 1S1R and destructive read operation issue of complementary resistive switching, demonstrating a self-selectivity of 10^10^ with an on/off resistance ratio larger than 10^3^ and an operation time constant of tens of nanoseconds in the heterostructure.

## Results

### Self-selective memory operation

**Fig. 1:**
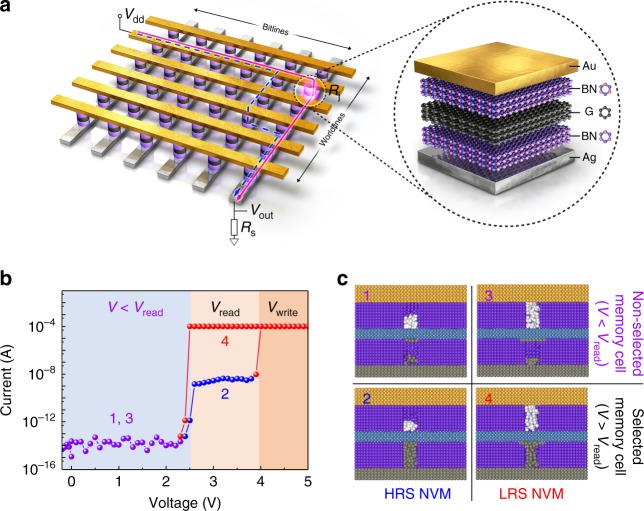
Crossbar memory array of a self-selective van der Waals heterostructure and the working mechanism. **a** Schematic picture of the van der Waals heterostructure in the crossbar memory array architecture, differing from the traditional one-selector one-resistor and complementary resistive switching (see in the main text). **b** Current–voltage characteristics of a single memory cell. The four current and voltage ranges represent four different states of the memory cell. The selectivity of the self-selective cell is 10^10^, with a big memory window (10^3^). Our unit cell shows bipolar behavior. For the negative voltage direction, it is shown in Supplementary Fig. 7a. During the measurement, the top electrode (gold) was kept to connect the ground. **c** Schematic illustration of hexagonal boron nitride/graphene/hexagonal boron nitride layers for the four states in ‘**b**’. Ranges “1” and “3” represent the high-resistance state and low-resistance state of unselected cells, respectively. Ranges “2” and “4” represent the high-resistance state and low-resistance state of a selected memory cell, respectively. Conductive silver filaments are formed at a voltage of 2.6 V, enabling the read of the high-resistance state (range “2”) and low-resistance state (range “4”) in a voltage window from 2.6 to 4.0 V. The gray, purple, blue, and yellow spheres represent silver, hexagonal boron nitride, graphene, and gold layer, respectively. The white spheres in the top hexagonal boron nitride layer represent the boron vacancies in hexagonal boron nitride

The self-selective van der Waals heterostructure memory cell, h-BN/graphene/h-BN, and its working mechanism with a possible sneak current path in a crossbar architecture are schematically described in Fig. 1a. The device fabrication steps and their optical image and Raman mapping are shown in the supplementary materials (Supplementary Figs. 1 and 2). Under the crossbar array structure with asymmetric Ag (for word lines) and Au (for bit lines) electrodes, the h-BN/graphene/h-BN structure is introduced at each intersection (Fig. 1a). A single memory cell exhibits characteristic memristive current–voltage (I–V) curves in positive voltage range as shown in Fig. 1b (a whole I–V curve ranging from negative to positive voltages is shown in Supplementary Fig. 7) where the voltage and current ranges are chosen to describe how the self-selective memory operates. Thus, the voltage and current ranges in Fig. 1b are categorized into four ranges: ranges “1” and “3” indicate the I–V performance of numerous unselected memory cells with inevitably applied voltages under a one-half (V/2) or one-third (V/3) voltage bias scheme, while ranges “2” and “4” indicate the I–V performance of a selected memory cell with a high-resistance state (HRS) and low-resistance state (LRS), respectively.

In Fig. 1b, a HRS/LRS conductance ratio of 10^3^ and a self-selectivity ratio of 10^10^ (defined as the resistance ratio of selected and unselected memory cells with probing voltages) are demonstrated in a single device operation. They are key features of our 2D self-selective cell, which is distinguished from former self-rectifying cells (see Supplementary Table 1).

The wide voltage windows for non-selective cells and the selected cells to enable a selectivity of beyond 10^10^ give sufficient voltage margins for various architectures and engineering. Moreover, the selected-state current (LRS and HRS) and threshold voltage could be tuned by the thickness of h-BN layers. The similar results on the thickness of switching layer-dependent switching behaviors have been reported in previously works^20,28^. We investigated a thinner h-BN based-device (Supplementary Fig. 3) showing a lower set voltage and on-state current; this enables tunable voltage and current for different applications. Further engineering for practical set voltages via controlling the thickness of h-BN layer is promising.

### Microscopic origin for the selectivity

High-resolution transmission electron microscopy (HR-TEM) was used to prove the unique dynamics for memory operation in the self-selective cell. We analyzed the cross-sectional images of a pristine memory cell and a memory cell after successive memory operation, a so-called ‘formed device’, by HR-TEM (Supplementary Figs. 4–6). Our in-situ TEM study could not produce reliable data due to the too large heat confinement and mechanical stresses applied to our device in the sample holder as similarly reported in the previous studies^29^. Instead, our ex-situ TEM study (see Supplementary Figs. 5 and 6), allows us to investigate more realistic device operation in our self-selective cell.

In the cross-sectional image of a ‘formed memory device’, we observed partial Ag layers between the graphene and bottom h-BN layer (Supplementary Fig. 5), which results from the migration of Ag ions through the h-BN layer during the memory operation; however, no Ag filament inside the bottom h-BN layer was observed in the ‘formed device’ by HR-TEM, indicating the volatile nature of the Ag filament at zero voltage bias. The volatile switching behavior of the unstable Ag filaments could be explained by the stable and strong in-plane atomic bonding of h-BN and the chemically inert interface between the h-BN and graphene layer. The discontinuous or broken state of the Ag filament at zero bias is kept until a voltage above 2.6 V is newly applied; below 2.6 V and without the Ag filaments, the high tunneling resistance of 15 nm thick h-BN generates low current states (with a resistance larger than 10^14^ Ω) in ranges “1” and “3” (Fig. 1b, c).

Once conductive Ag filaments are formed in the h-BN layer between the Ag electrode and graphene by a newly applied voltage larger than 2.6 V, the total memory resistance is dominated by the presence of conducting paths formed by the migration of boron vacancies in the h-BN layer between the Au electrode and graphene; a low activation energy and non-volatile memory behavior of the boron vacancies without Ag electrodes have been reported^30,31^. A defective path that can act as a boron vacancy filament was observed between the Au electrode and graphene (see Supplementary Fig. 6). It has been reported that such conducting paths through boron vacancies show non-volatile transport via trap-assisted space charge limited conduction that can be used as NVM;^30,31^ thus, ranges “2” and “4” in Fig. 1b, c indicate the non-volatile HRS and LRS. Finally, a voltage larger than 4 V converts the HRS to the LRS by creating non-volatile conducting paths composed of boron vacancies; these non-volatile conducting paths can be broken by applying an opposite voltage bias (−4 V) in the reset process (Supplementary Fig. 7a)^28^. The corresponding electroforming processes are shown in Supplementary Fig. 8.

### Memory integration with low sneak current

**Fig. 2:**
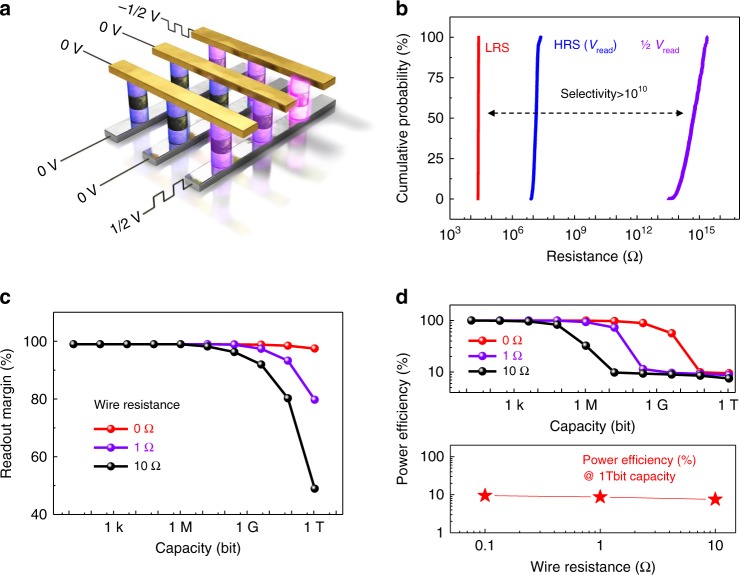
Memory integration of self-selective memory cells. **a** Schematic picture of a reading process using a one-half voltage scheme. The selected memory cell with a net voltage application of ‘V’ is highlighted in pink, while either one-half ‘V’ or zero voltage bias is applied to the other memory cells. **b** Reliability of the three states: the low-resistance state (probed by V_read_), high-resistance state (probed by V_read_), and unselected state (probed by one-half V_read_) exhibit narrow voltage windows and a high selectivity of larger than 10^10^ in the cumulative probability of resistances. **c** Readout margin for three different wire resistances between neighboring cells simulated by using SPICE modeling. A 1/2 V voltage scheme was used in the simulation, while a 1/3 V voltage scheme showed a similar result (Supplementary Fig. 11). **d** Simulated capacity-dependent energy efficiency with three different wire resistances. An energy efficiency of 10% was observed at an integration capacity of one terabit by SPICE modeling, which is consistent with the greatly suppressed sneak current due to the high selectivity of larger than 10^10^ in (**b**). The unit wire resistance calculated in our work is less than 10 Ω (see Methods). Thus, the maximum wire resistance used for this simulation is 10 Ω

The memory performance of integrated self-selective van der Waals cells in the crossbar array was evaluated using a one-half (V/2) bias voltage scheme, as shown in Fig. 2. The schematic illustration of a self-selective memory crossbar array with a selected memory cell (highlighted in pink color) in Fig. 2a describes a reading operation with voltage application in a V/2 bias voltage scheme; a net voltage of “V” is applied to the selected cell. Among the eight unselected cells, four cells (highlighted by a light clear pink color) still experience a voltage of “V/2”, while the other four cells (violet color) have zero applied voltage. According to the single cell performance is shown in Fig. 1, the eight unselected cells have applied voltages below the threshold voltage of 2.6 V and thus possess a resistance larger than 10^14^ Ω. The reliability of the unselected cells, indicated by the cumulative probability of the HRS, is demonstrated in Fig. 2b. The non-volatile LRS and HRS also exhibit stable operation (steady resistances of LRS and HRS in Fig. 2b). To show the statistic performance, the cumulative probability of HRS, LRS based on the device to device variation (144 devices) is shown in Supplementary Figs. 9 and 10.

Based on the performance of a single self-selective cell, a SPICE model was built to explore the evolution of readout margin and energy efficiency as we increase the integration capacity with our self-selective memory cells. The current vs. voltage curve over the full-operation voltage range (from −5 to + 5 V) was simulated first to determine the validity of our SPICE simulation (the fitted I–V curve and the reading bias voltage scheme are shown in Supplementary Fig. 7a). Then, the readout margin and energy efficiency of the integrated cells are simulated by SPICE modeling (Fig. 2c, d). The readout margin in Fig. 2c demonstrates a value close to 100% up to megabit-scale (10^6^) capacity with variable inter-cell wire resistances. We note that practical readout margin is kept until terabit-scale integration in Fig. 2c; the readout margin is above 45% at a terabit (10^12^) capacity even with a wire resistance of 10 Ω (10 Ω is enough for our case, see Methods), larger than the acceptable value (10%)^32,33^. Therefore, the LRS and HRS of memory can be easily distinguished with an effective margin for terabit-scale integration. The one-third (V/3) voltage scheme exhibits similar results (Methods and Supplementary Fig. 11). Further advanced synthesis technique of graphene and h-BN could realize the large-scale integration.

The influence of sneak current can be directly quantified by comparing the power consumption in the selected and all other unselected memory cells. Accordingly, the capacity-dependent power consumption with different wire resistances in integrated memory cells is simulated by SPICE modeling, as shown in Fig. 2d. Even at a one terabit-scale, the power consumption ratio reaches 10% with the wire resistance set as 10 Ω; one selected cell consumes 10% of the total power that the total 10^12^ cells consume. In other words, the sneak current is greatly suppressed with our self-selective van der Waals structure. We note that state-of-the-art power efficiency at an array size of 10^5^ remains only 0.02%^5^. This finding is consistent with the selectivity of beyond 10^10^ described in Fig. 1b, demonstrating that our self-selective memory cells effectively suppressed sneak currents, which cannot be demonstrated by other conventional devices (Supplementary Table 1).

**Fig. 3:**
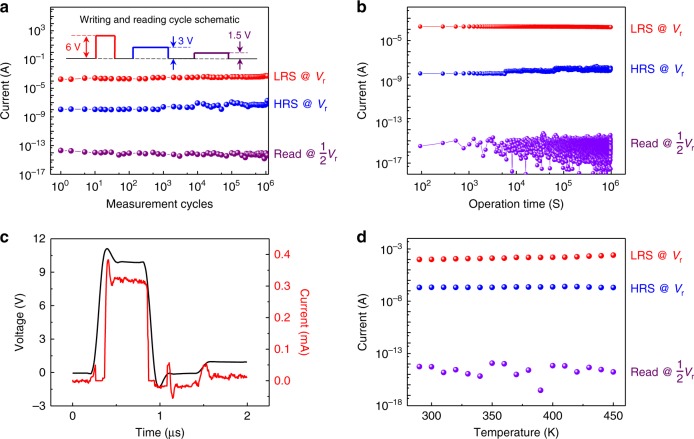
Stability and switching speed of our self-selective memory. **a** Endurance of switching behavior among the three states by a voltage pulse train over 10^6^ measurement cycles. The resistance states were read by a wide pulse width to avoid the net charge (See Methods). **b** Retention behavior of the three states for a time of 10^6^ s. **c** Switching speed during programming operation shows a time constant of tens of nanoseconds. A voltage pulse of 10 V for 500 ns was used for the measurement. **d** Stability of the three states (low-resistance state, high-resistance state, and unselected state) over temperatures ranging from 290 to 450 K, due to the high crystal quality of hexagonal boron nitride and graphene

The endurance and reliability of volatile and NVM behaviors in our self-selective memory cells were examined over 10^6^ write-and-reset cycles, and the results are summarized in Fig. 3a. Our device is much more stable than previously reported 2D materials-based memory cells with limited switching cycles of hundreds of cycles^19,20,28,34,35^. A voltage pulse of 6 V (−6 V) for a time duration of 0.1 ms was used to set from the HRS to the LRS (reset from the LRS to the HRS) in the cell. A reading voltage of 3 V and a smaller voltage (1.5 V under a one-half voltage bias scheme) for the unselected cell state followed each write-and-reset process. Steady currents for the LRS (red dots), HRS (blue dots), and one-half reading voltage (violet dots) over 10^6^ cycles were observed, as shown in Fig. 3a. In addition, we confirmed that all three states are stable over 10 ^6^ s (Fig. 3b), originating from the van der Waals heterostructure with the two most stable 2D elements (h-BN and graphene).

Switching speed between memory states is a key factor in a highly integrated self-selective memory array outperforming the current flash memory technology^7^. The red curve in Fig. 3c exhibits a typical transient switching behavior with a time constant of tens of nanoseconds in our self-selective cell by a writing voltage pulse. After the pulse, the zero voltage bias makes the memory cell be in a non-selected state within a similar time-scale (<50 ns). Based on the results shown in Fig. 3c, we can also estimate the energy for ‘write’ operation as 1.5 nJ (the pulse width, amplitude, and current are 0.5 μs, 10 V, and 0.3 mA, respectively), which is almost 1000 times smaller than those of flash memory^36^. The current density of our device is ~3 × 10^4^ A/cm^2^ (ON-state current divided by device area around 1 μm^2^), which is much lower than that of conventional flash memory (10^6^ A/cm^2^)^37^. We note that the operation voltage and current can be optimized through process and device engineering to further reduce energy consumption. Another important factor for practical applications of memory arrays is device stability over a wide range of temperatures: due to the highly stable properties and non-sensitive to ambient of h-BN^24,38^, increased temperatures in real applications or extreme temperature environments produce steady currents or memory states in our self-selective memory cells, as shown in Fig. 3d.

### Demonstration of self-selective memory array with 2D materials

**Fig. 4:**
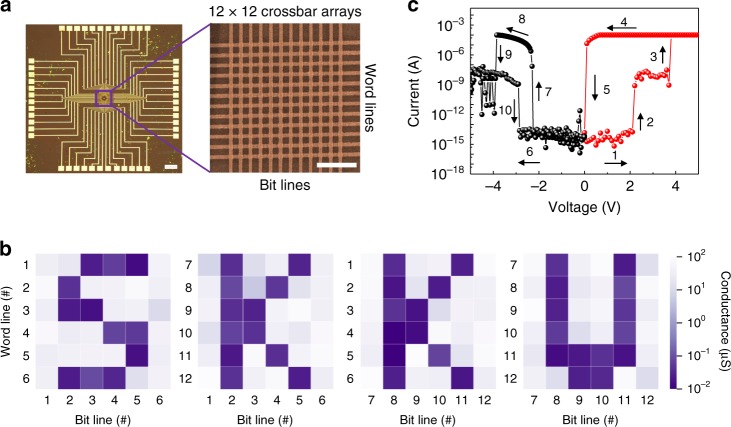
Programming a code using a crossbar array with our self-selective memory cells. **a** Optical and scanning electron microscopy images of a 12 × 12 crossbar memory array with our self-selective van der Waals heterostructure experimentally. Scale bars: 200 and 5 μm for the optical and SEM images, respectively. **b** The color map of the readout conductance with a reading voltage of 3 V (one-half voltage scheme). The heavy violet color indicates a lower readout conductance, as shown in the conductance scale. **c** A full-voltage-range resistive switching curve of a self-selective cell fabricated on flexible PET substrate. The sequential numbers and arrows exhibit typical write and erase processes, maintaining the negligible sneak current for unselected memory cells

Programming and reading 144 binary bits in a 12 × 12 crossbar array of our self-selective memory cells within lateral dimensions of 15 × 15 μm^2^ are demonstrated as a proof-of-concept in Fig. 4. Given the large selectivity of 10^10^ of the memory cell, all three conventional programming schemes, namely, floating, one-half voltage, and one-third voltage schemes, can be applied in the memory crossbar array with a sufficient readout margin and energy efficiency. In Fig. 4, as an example, a 5 V writing voltage was chosen. Thus, only the selected cell is programmed, while the current flowing through all other unselected cells is kept at an extremely low level (below 10 fA). We note that the ultralow sneak current (10 fA) is also maintained during the reading operation.

Based on the 12 × 12 crossbar arrays of our self-selective memory cells, a code of “SKKU” was programmed using 144 binary bits for four letters (SKKU), as shown in Fig. 4b. Each intersection of a bit line and a word line has an address number and a non-volatile conductance as the binary information (Fig. 4b), which can be combined to register the intended code. Moreover, the self-selective memory operation was validated on a flexible substrate, polyethylene terephthalate (PET) (Supplementary Fig. 12). The full operation of a single memory cell on PET over a voltage sweep following sequential numbers and arrows is shown in Fig. 4c. The I–V curve in Fig. 4c is similar to the full operation curve (Supplementary Fig. 7a); in the measurements, a compliance current of 10^–4^ A was applied for reliable operation, and a reading voltage window ranging from 2.3 to 3.7 V and a writing voltage window (>4 V) were observed along the sequential numbers and arrows (Fig. 4c).

## Discussion

Our self-selective memory based on a van der Waals heterostructure (h-BN/graphene/h-BN) resolves a fundamental issue, sneak currents, over a large voltage window, which overcomes former memristive devices (e.g., 1S1R, 1T1R, or complementary resistive switching). This advancement opens a door for large-scale and energy-efficient memory integration with a wafer-scale growth of few-layered high quality h-BN. The unique stability and impermeability of graphene and h-BN provide breakthroughs, such as atomic valves for Ag ions and the boron vacancy kinetics in h-BN, for the novel and stable memory operation. Thus, our new memory device allows efficient crossbar memory arrays on flexible substrates for future memory applications, such as the internet of things and wearable artificial intelligence.

## Methods

### Device fabrication procedures

The 2D materials (graphene and hBN) are transferred on a silicon substrate with a 300 nm dry oxide layer (SiO_2_) on its top surface. HOPG (highly ordered pyrolytic graphite) crystal was purchased from HQ Graphene and h-BN crystal was obtained from Prof Takashi Taniguchi’s group at the National Institute of Materials Science, Japan. First, the bottom electrode (BE, Ag) with a pre-designed pattern was deposited on the Si/SiO_2_ substrate. Next, the h-BN flake was exfoliated on the polydimethylsiloxane (PDMS) substrate and transferred onto the BE by a typical dry-transfer technique. Then, the graphene layer exfoliated on the prepared PDMS substrate was transferred onto the h-BN layer with a smaller size than that of the h-BN layer below. The same transfer technique was used again to transfer another h-BN layer on top of the graphene layer. Then, only the h-BN and graphene layers at the target united cell area were kept, and all other parts were etched by RIE. Finally, gold was deposited by a thermal evaporator to form the top electrode (TE) in the pre-designed area. During the dry-transfer technique, the layer-by-layer alignment was assisted by optical microscopy.

### Device patterning details

The patterning of both TE and BE was designed by electron beam lithography (EBL). First, the silicon substrate with a 300 nm dry oxide layer was spin-coated with poly (methylmethacrylate) (PMMA) 950 A4. The spin speed was 500 rpm for the first 5 s and it was increased to 4000 rpm for 60 s. Then, the sample was baked at 120 °C on a hot plate for 2 mins. Next, EBL was used to expose the pre-designed electrode pattern, and then a typical developing process was employed. Finally, silver (Ag) deposition was conducted by a thermal evaporator, followed by a 2 h lift-off process in acetone. Then, the dry-transfer technique was used to transfer the heterojunctions on the prepared Ag electrode. After that, the same EBL patterning processing as mentioned above was used for patterning the TE (Au). The metal deposition method for the Au electrode was thermally evaporated. For the flexible device, photolithography was used to fabricate the patterns on a PET substrate with LOR 2 A and AZ GXR-601 as double-layered photoresists (PRs) as follows: (1) LOR 2 A (MicroChem Corp) was spin-coated on the PET substrate (500 rpm for 5 s and then 3000 rpm for 30 s), followed by AZ GXR-601 (AZ electronic materials Co. Ltd) with the same spin speed. Then, the sample was baked on a hot plate at 110 °C for 1 min. (2) The sample was exposed under UV light for 10 s with a mask aligner machine and photo mask. (3) The sample was dipped into the AZ 300 MIF developer (AZ Electronic Materials Co. Ltd) for 20 s to remove the exposed PR. (4) Metal deposition of Ag with a thickness of 50 nm was performed, followed by lift-off with AZ 100 remover (AZ Electronic Materials Co. Ltd.). (5) The heterostructure (h-BN/graphene/h-BN) was transferred onto the pre-designed Ag electrode. (6) The same photolithography processes were conducted with the TE materials as Au at 50 nm.

### Optical characterization

Raman spectra and images were measured with a Witec Alpha300 confocal system located in a glove box to avoid the effect of H_2_O or O_2_ on the measurements. The concentrations of H_2_O or O_2_ were 0.6 ppm and 0 ppm, respectively. The laser wavelength for both the Raman spectra and images was 532 nm, and the grating used for the measurement was 600 mm^−1^. The integration time for single spectra and images were 10 s and 0.2 s, respectively. The laser power was kept below 0.5 mW to avoid heating effects on the samples. A 100x objective lens (Zeiss) was used to focus the laser beam.

### Key electrical measurement conditions

Electrical measurements were performed at room temperature in vacuum, using a Keithley 4200 semiconductor parameter analyzer and a Cascade probe station. Two modules were used: a direct current (DC) source unit (4200-SMU) with high precision pre-amplifiers and a 4225-PMU waveform generator unit for pulse measurement. During DC and pulse testing, the positive voltage output was connected to the Ag (BE). The Au (TE) was kept as the ground electrode. During the pulse testing, the maximum current range was set to 10 mA, which sets the current resolution at 1 µA. Initially, all devices were at high resistance in the initial state and were SET to the LRS with a 100 µs/6 V pulse and RESET to the HRS with a 100 µs/-6 V pulse. The resistance states were read with 5 ms/± 3 V pulses that cannot change the device state but just open the off-state. The RESET and SET voltages are higher than those measured with DC sweeps (VSET = 4 V, VRESET = −3.8 V at DC).

### SPICE modeling

The resistive switching curve of the self-selective memory device based on a van der Waals heterojunction (h-BN/graphene/h-BN) shown in Fig. 1b was modeled using Verilog-A. The large-scale crossbar array structure based on this device was simulated using Cadence Spectre. Detailed descriptions of device modeling and large-scale array simulation are provided (Supplementary Figs. 7 and 11).

As shown in Supplementary Fig. 7, two kinds of bias voltage schemes were used in this simulation. (a) The one-half voltage bias scheme: The voltage is applied across the selected word and bit line, while all other bit and word lines are applied with a half voltage bias. As shown in Supplementary Fig. 7b, there will be no bias voltage applied to the cells in the gray-colored background. In contrast, the cells within the magenta-colored area are half-voltage biased. In our self-selective van der Waals heterostructure-based memory cells, the sneak path current can be dramatically suppressed to less than 10^−14^ A for half-biased cells due to extreme non-linearity and high-selectivity behaviors. (b) The one-third voltage bias scheme: in this case, the selected word line is fully biased, while the selected bit line is grounded. Then, the unselected word lines and bit lines are biased at 1/3 and 2/3 of the full bias voltage, respectively. Therefore, the cells within the gray-colored area are under one-third of the full bias voltage, and the cells in the magenta-colored area are under one-third of the full bias voltage.

In this simulation, the wire resistance is selected as 0 Ω, 1 Ω, or 10 Ω, respectively. In our work, the wire resistance is much less than 10 Ω. The evaluation details are as below: supposing that the feature size is F, L = 2 F, and S = F*T, where T denotes the wire thickness (in our work, the thicknesses of both the Ag and Au layers are 50 nm). The unit wire resistance = ρL/S, and the resistivity of Ag and Au is 15.87 nΩ m, and 22.14 nΩ m, respectively. This gives the resistance of Ag and Au as 0.635 Ω and 0.88 Ω, respectively, which are much lower than 10 Ω.

## Supplementary information

Supplementary Information

## Data Availability

All data needed to evaluate the conclusions in the paper are present in the paper and/or the Supplementary Materials. Additional data related to this paper are available from the authors on reasonable request; see author contributions for specific data sets.
