# Supplementary material for: Self-selective van der Waals heterostructures for large scale memory array
Source: Nat Commun. 2019 Jul 18;10:3161. doi: 10.1038/s41467-019-11187-9 (PMC6639341; doi:10.1038/s41467-019-11187-9)
Supplement: Supplementary file 1 — Supplementary Information [file 41467_2019_11187_MOESM1_ESM.pdf]

Supplementary Information

Self-selective van der Waals heterostructure for large scale memory array

By Sun et al.

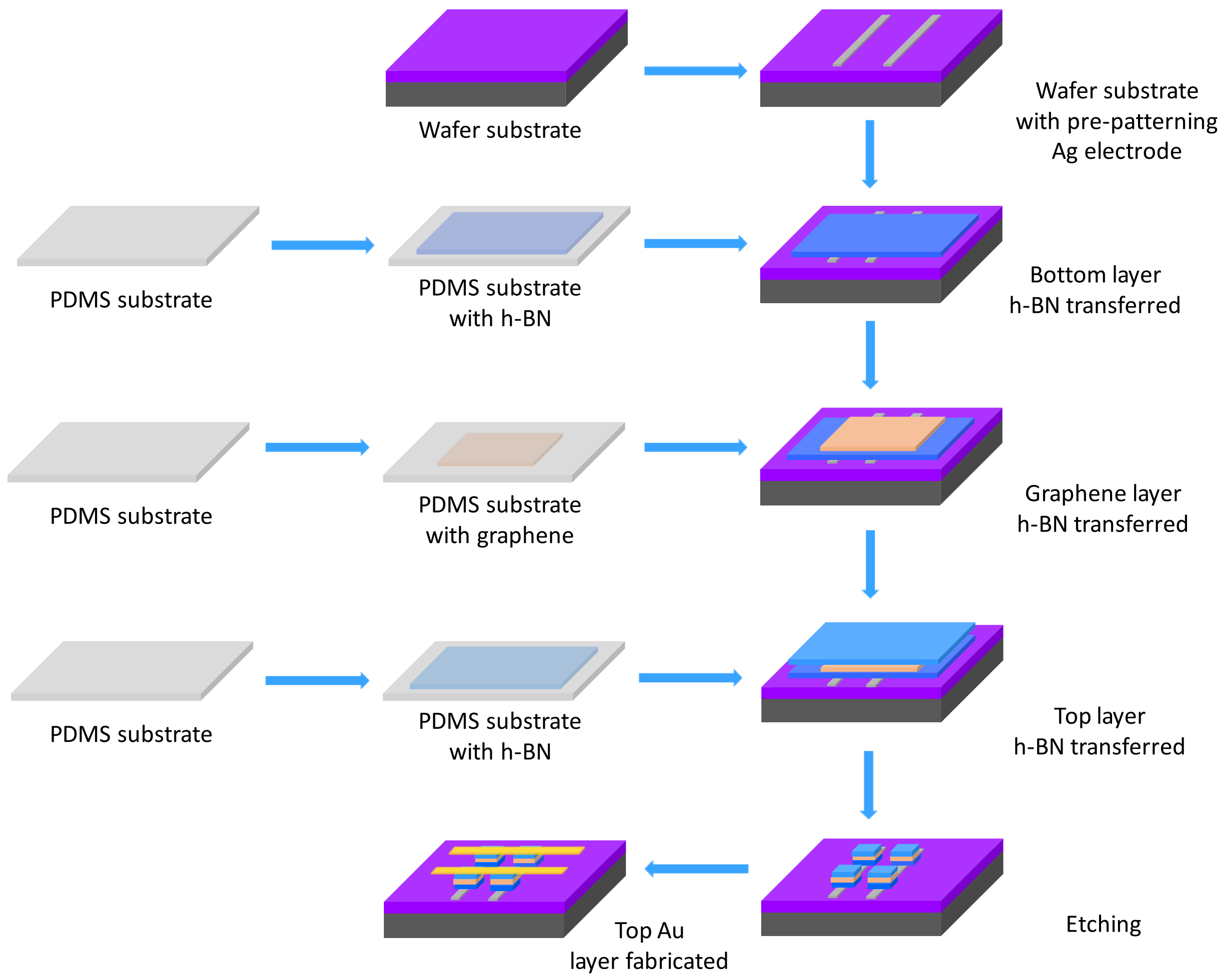

Supplementary Figure 1 Schematic illustration of device fabrication steps. (1) First, the Si/SiO_2 substrate is patterned with Ag as the bottom electrode for our self-selective memory cells by using e-beam lithography and lift-off processes (see details in Methods: Device fabrication). Then, we prepare a polydimethylsiloxane (PDMS) substrate with a pre-exfoliated h-BN layer on it and transfer the suitable h-BN layer on the pre-pattern Ag electrode, assisted by optical microscopy. (2) Using the same methods, another graphene layer is transferred onto the pre-transferred h-BN layer. The alignment during the transfer processes is assisted by optical microscopy. (3) Another h-BN layer with a small thickness is transferred on top of the structure discussed above. (4) Etching the area of the 2D layered materials without the Ag electrode support, as shown in the figure above. (5) Finally, the Au electrode is patterned, forming a cross bar shape relative to the bottom Ag electrode. This schematic is viable for only the memory cell array measurement. For the measurement of a single device, the etching step shown above was omitted (Figure 1 and Supplementary Figure 2).

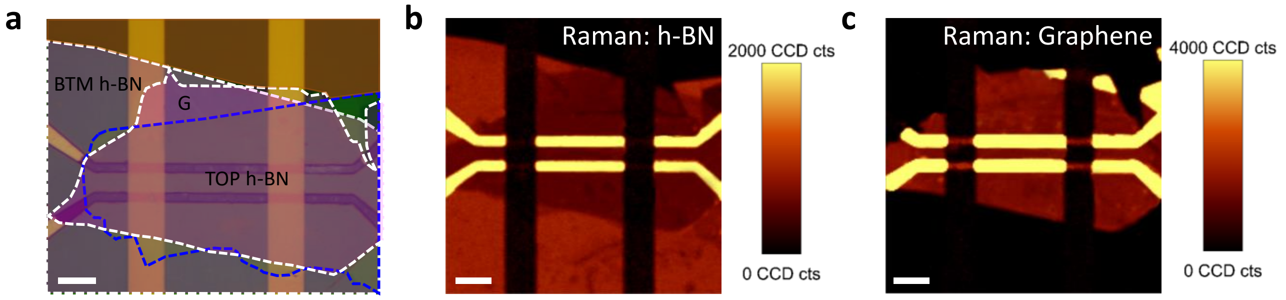

Supplementary Figure 2 Material characterization of van der Waals structures. **a,** Optical image of the fabricated 2×2 self-selective memory. We selected one of them as a single device to measure the I-V curve. In this image, the narrow electrode is Ag while the wide electrode is Au. The areas marked using yellow, white and blue dashed lines represent the bottom h-BN, graphene and top h-BN layers, respectively. The scale bar is 10 μm. **b** and **c** Raman mapping images integrated with the Raman peaks from G bands in h-BN and G band in graphene, respectively. The scale bar is 10 μm.

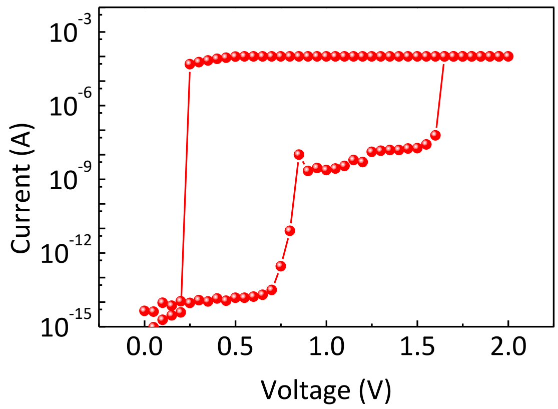

Supplementary Figure 3 Typical I-V curve using thin h-BN layer (~5nm), with a clearly decreased Vth.

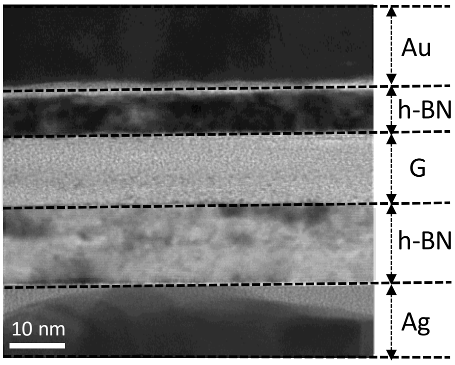

Supplementary Figure 4 Cross-sectional TEM image of our self-selective devices. From top to bottom, there are five layers: Au, thin h-BN, graphene, thick h-BN and Ag layers.

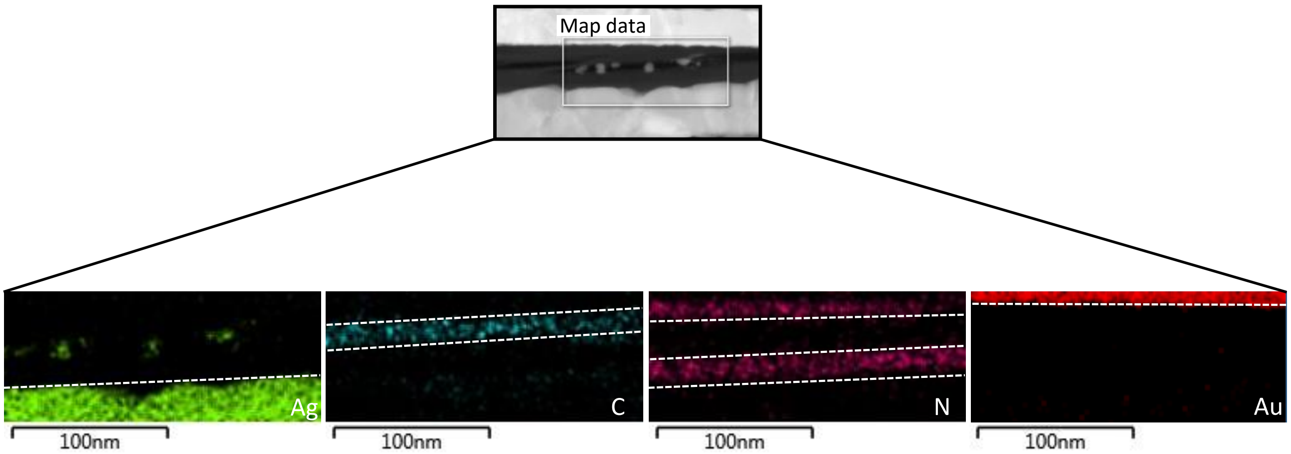

Supplementary Figure 5 The EDX mapping for all involved elements, including the van der Waals structures and electrodes. The clear silver particles are shown near the interface between graphene and bottom the h-BN layer, but there are no sliver particles observed between the Au and top h-BN layer, which is consistent with the discussion in the main text.

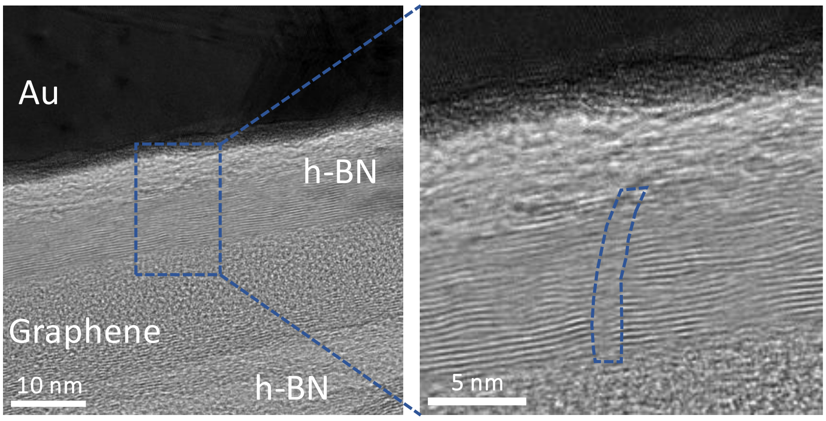

Supplementary Figure 6 The observed defective path in the h-BN layer stacked between the Au and graphene layers, which functions as NVM, as shown by the dashed line in the right zoomed-in figure.

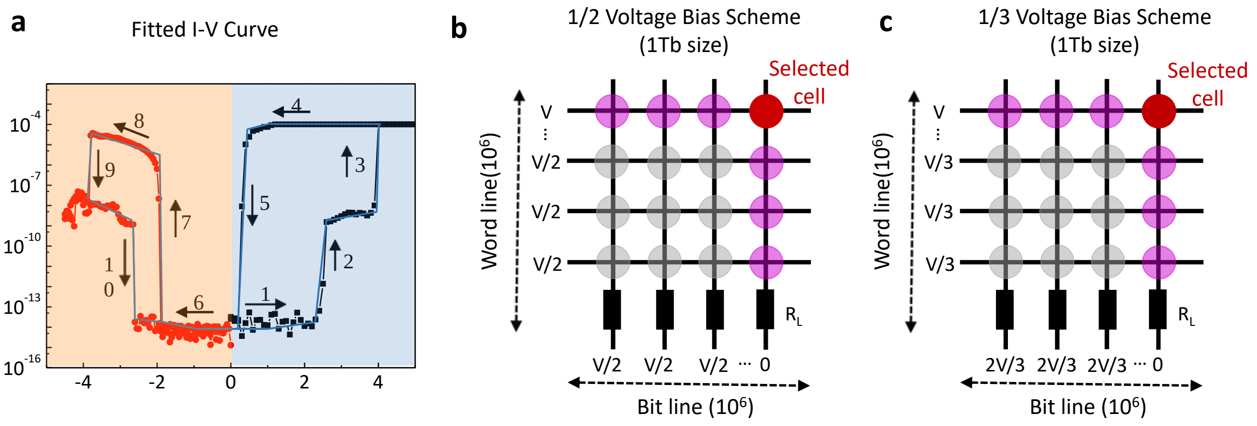

Supplementary Figure 7 The fitted curve for simulation and two bias voltage schemes used in this work. **a,** the simulated DC I-V curve (solid straight line) based on a single self-selective memory cell from the SPICE simulation. **b,** 1/2 voltage bias scheme; **c,** 1/3 voltage bias scheme. For a detailed description of the difference between these two kinds of bias scheme, please refer to the Methods.

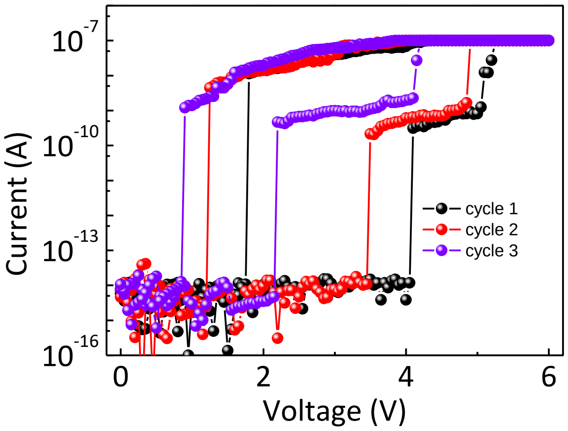

Supplementary Figure 8 The electroforming processes for the self-selective memory cells. Three typical cycles during switching are shown.

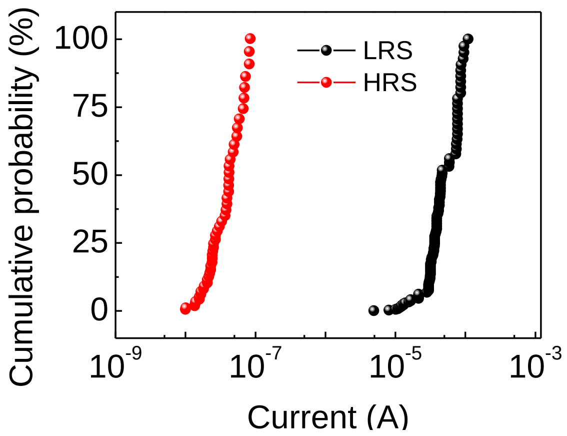

Supplementary Figure 9 Device-to-device variations of HRS and LRS tested on 144 devices.

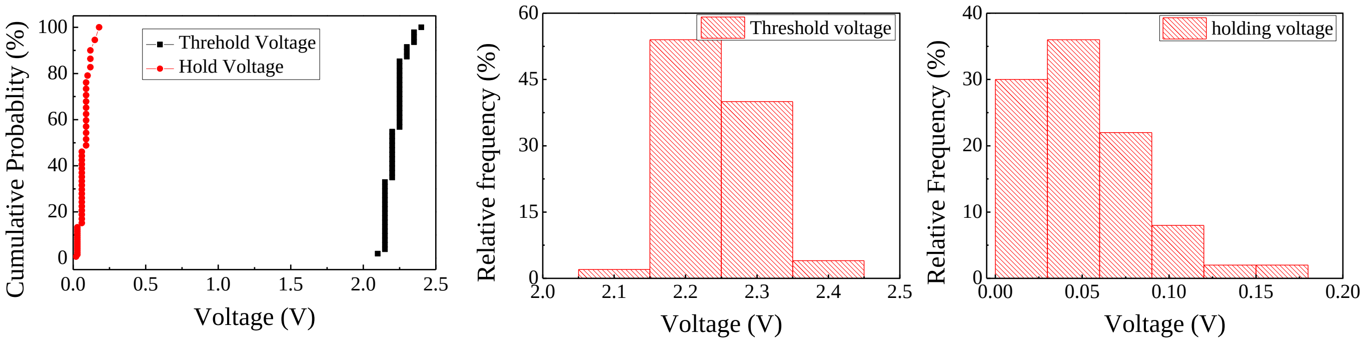

Supplementary Figure 10 Cumulative probability and histogram of threshold and holding voltage. Once the device is formed (as shown in Supplementary Figure 7a), our devices show a reliable threshold and holding voltage.

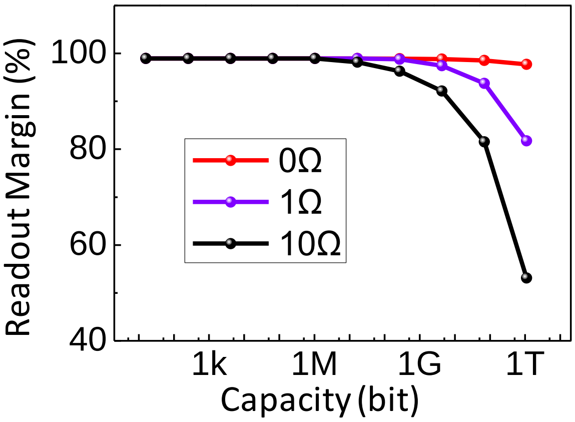

Supplementary Figure 11 Readout margin for three different wire resistances between neighboring cells simulated by using SPICE modelling. A one-third voltage scheme was used for this simulation showing a similar result to that obtained using a one-half voltage scheme (Figure 2c). The red, purple and black curves indicates wire resistances of 0 Ω, 1 Ω, 10 Ω, respectively.

a 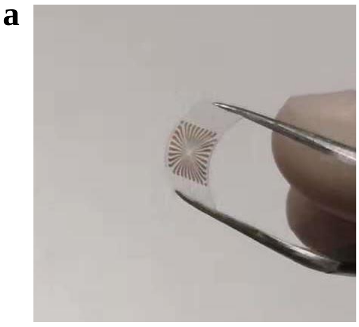 **b** 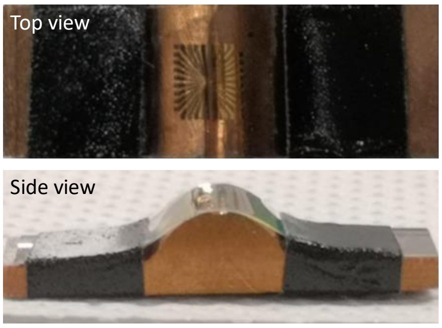

Supplementary Figure 12 Optical image of a flexible device on a PET substrate. **a**, an optical image of a device fabricated on flexible substrate. **b**, the top view and side vide of set-up for measurement. A typical device characteristic curve on the bent substrate is shown in Figure 4c (main text).

Structure	ON/OFF ratio	Rectification ratio	Off current	Endurance	Retention	Polarity	Ref
Cu/HfO_2/n-Si	10^4 @1 V	10^4 @±1V	1 nA@1V	50 (DC)	2000 s@85℃	unipolar	[1]
Pt/NbO_x/TiO_y/NbO_x/TiN	10^2 @3V	10^5	1 pA @3V	5000 (DC)	10 yrs	bipolar	[2]
p-Si/SiO_2/n-Si	>10^4 @1.5 V	10^5 @±1.5 V	10 nA @2 V	>100 (DC)	>2×10^5 s @ 300℃	unipolar	[3]
W/Al_2O_3/TaO_x/TiN	>10	>5000	1 nA @0.65 V	10^8	>900h@ 85℃	bipolar	[4]
Pt/Ta_2O_5/HfO2/TiN	10^3 @6 V	10^4 @6 V	>10 pA@±5V	10^3 (DC)	10^46@85℃	bipolar	[5]
Pd/HfO_2/WO_x/W	>10	100 @0.35 V	100 nA@-1 V	10^9	10^4 s @85℃	bipolar	[6]
Ti/SiO_xN_y/AIN/Pt	80	10^2	200 nA @±2 V	10^3	10^5 s	bipolar	[7]
Ni/HfO_2/n^+-Si	10^4 @0.1 V	10^3 @ 1V	>10 pA @±0.5 V	100 (DC)	>2×10^4 s @ 125℃	unipolar	[8–10]
Si/a-Si core/Ag nanowires	10^4	10^6 @±1.5V	1 pA @±1.5 V	10^4	>2 weeks	bipolar	[11]
Pt/Ti/Ta_2O_{5-x}/TaO_{2-x}/ Pt	10	10^5 @±0.7	10 μA @-1 V	10^9	NA	bipolar	[12]
Ag/h-BN/Graphene/ h-BN/Au	10^4	10^{10}	10 fA @±1.5V	10^4	10^6 s	bipolar	This work

Structure	Off current	On/off ratio	selectivity	Two-terminal	ref
Pt/Ag/SiO_x:Ag/Pt/Pd/Ta/Ta_2O_5:Ag/Ru	10 pA	>10	10^7	no	[13]
Ag/Defect graphene/SiO_2/Pt/HfO_2/Cu	1 pA	>100	10^9	no	[14]
Mg/MgO/W/Ag/MgO/Ag/W	10 pA	>100	10^7	no	[15]
Pt/HfO_x/Ag/W/NbOx/W	1 pA	100	10^7	no	[16]
Ti/ZrO2/Pt/Ag/ZrO2:Ag/Pt	1 nA	10	105	no	[17]
Pt/Ag/HfO_x/Ag/Pd/TaOx/Ta_2O_5/Pd	>1 pA	10	10^7	no	[18]
Ag/h-BN/graphene/h-BN/Au	10 fA	10^4	10^{10}	yes	This work

Supplementary Table 1 Comparison of the performance of previously reported NVM devices, including self-rectifying memristors (top table) and non-self-rectifying memristors (bottom table). It is clearly shown that compared with that of non-self-rectifying cells, the operation performance of self-rectifying memory cells is usually poor, although their device structure is relatively simple. However, the complex device structure, fabrication processes, and the three-terminal architectures for non-self-rectifying memory cells hinder their application for 3D integration. Our new self-selective memory cells possess the advantages of both self- and non-self-rectifying memory cells.

Supplementary References

1 Wang, M. J., Gao, S., Zeng, F., Song, C. & Pan, F. Unipolar resistive switching with forming-free and self-rectifying effects in Cu/HfO_2 /n-Si devices. *AIP Adv.* 6, 025007 (2016).

2 Kim, K. M. et al. Low-Power, Self-Rectifying, and Forming-Free Memristor with an Asymmetric Programing Voltage for a High-Density Crossbar Application. *Nano Lett.* 16, 6724–6732 (2016).

3 Li, C. et al. Three-dimensional crossbar arrays of self-rectifying Si/SiO_2/Si memristors. *Nat. Commun* 8, 15666 (2017).

4 Samanta, S., Maikap, S., Roy, A., Jana, S. & Qiu, J.T. Effects of W/Ir Top Electrode on Resistive Switching and Dopamine Sensing by Using Optimized TaO_x-Based Memory Platform. *Adv Mater Interfaces* 4, 1700959 (2017).

5 Yoon, J. H. et al. Highly Uniform, Electroforming-Free, and Self-Rectifying Resistive Memory in the Pt/Ta_2O_5/HfO_{2-x}/TiN Structure. *Adv Funct Mater.* 24, 5086–5095 (2014).

6 Luo, Q. et al. Self-Rectifying and Forming-Free Resistive-Switching Device for Embedded Memory Application. *IEEE Electron Device Lett* 39, 664–667 (2018).

7 Kwon, J. Y., Park, J. H. & Kim, T. G. Self-rectifying resistive-switching characteristics with ultralow operating currents in SiO_xN_yAlN bilayer devices. *Appl. Phys. Lett* 106, 223506 (2015).

8 Rodríguez-Fernandez, A. et al. Resistive Switching with Self-Rectifying Tunability and Influence of the Oxide Layer Thickness in Ni/HfO_2/n+-Si RRAM Devices. *IEEE Trans. Electron Devices* 64, 3159–3166 (2017).

9 Lu, D. et al. Investigations of Conduction Mechanisms of the Self-Rectifying n+Si-HfO_2–Ni RRAM Devices. *IEEE Trans. Electron Devices* 61, 2294–2301 (2014).

10 Tran, X. A. et al. Self-rectifying and forming-free unipolar HfO_x based-high performance RRAM built by fab-avaialbe materials. in *2011 International Electron Devices Meeting* 31.2.1-31.2.4 (2011).

11 Dong, Y., Yu, G., McAlpine, M. C., Lu, W. & Lieber, C. M. Si/a-Si Core/Shell Nanowires as Nonvolatile Crossbar Switches. *Nano Lett.* 8, 386–391 (2008).

12 Lee, M.-J. et al. A fast, high-endurance and scalable non-volatile memory device made from asymmetric Ta_2O_{5-x}/TaO_{2-x} bilayer structures. *Nat. Mater* 10, 625–630 (2011).

13 Yoon, J. H. et al. Truly Electroforming-Free and Low-Energy Memristors with Preconditioned Conductive Tunneling Paths. *Adv. Funct. Mater* 1702010 (2017).

14 Zhao, X. et al. Breaking the Current-Retention Dilemma in Cation-Based Resistive Switching Devices Utilizing Graphene with Controlled Defects. *Adv.Mater* 30, 1705193 (2018).

15 Sun, J. et al. Physically Transient Threshold Switching Device Based on Magnesium Oxide for Security Application. *Small* 1800945 (2018).

16 Park, J., Yoo, J., Song, J., Sung, C. & Hwang, H. Hybrid Selector with Excellent Selectivity and Fast Switching Speed for X-point memory array. *IEEE Electron Device Lett* 1 (2018).

17 Wang, C., Song, B. & Zeng, Z. Excellent selector performance in engineered Ag/ZrO_2:Ag/Pt structure for high-density bipolar RRAM applications. *AIP Adv.* 7, 125209 (2017).

18 Midya, R. et al. Anatomy of Ag/Hafnia-Based Selectors with 10^{10} Nonlinearity. *Adv. Mater* 29, 1604457 (2017).
